# Supplementary material for: Do Protected Areas Matter? A Systematic Review of the Social and Ecological Impacts of the Establishment of Protected Areas
Source: Int J Environ Res Public Health. 2020 Oct 4;17(19):7259. doi: 10.3390/ijerph17197259 (PMC7579073; doi:10.3390/ijerph17197259)
Supplement: Supplementary file 1 [file ijerph-17-07259-s001.pdf]

List of articles covered in this study

[1] Andam, K. S., Ferraro, P. J., & Hanauer, M. M. (2013). The effects of protected area systems on ecosystem restoration: a quasi-experimental design to estimate the impact of Costa Rica's protected area system on forest regrowth. Conservation Letters, 6(5), 317-323.

[2] Andam, K. S., Ferraro, P. J., Sims, K. R., Healy, A., & Holland, M. B. (2010). Protected areas reduced poverty in Costa Rica and Thailand. Proceedings of the National Academy of Sciences, 107(22), 9996-10001.

[3] Blackman, A., Pfaff, A., & Robalino, J. (2015). Paper park performance: Mexico's natural protected areas in the 1990s. Global Environmental Change, 31, 50-61.

[4] Canavire-Bacarreza, G., & Hanauer, M. M. (2013). Estimating the impacts of Bolivia’s protected areas on poverty. World Development, 41, 265-285.

[5] Clements, T., Suon, S., Wilkie, D. S., & Milner-Gulland, E. J. (2014). Impacts of protected areas on local livelihoods in Cambodia. World Development, 64, S125-S134.

[6] den Braber, B., Evans, K. L., & Oldekop, J. A. (2018). Impact of protected areas on poverty, extreme poverty, and inequality in Nepal. Conservation Letters, 11(6), e12576.

[7] Duan, W., & Wen, Y. (2017). Impacts of protected areas on local livelihoods: Evidence of giant panda biosphere reserves in Sichuan Province, China. Land Use Policy, 68, 168-178.

[8] Ferraro, P. J., & Hanauer, M. M. (2011). Protecting ecosystems and alleviating poverty with parks and reserves: ‘win-win’ or tradeoffs? Environmental and resource economics, 48(2), 269-286.

[9] Ferraro, P. J., Hanauer, M. M., & Sims, K. R. (2011). Conditions associated with protected area success in conservation and poverty reduction. Proceedings of the National Academy of Sciences, 108(34), 13913-13918.

[10] Ferraro, P. J., Hanauer, M. M., Miteva, D. A., Canavire-Bacarreza, G. J., Pattanayak, S. K., & Sims, K. R. (2013). More strictly protected areas are not necessarily more protective: evidence from Bolivia, Costa Rica, Indonesia, and Thailand. Environmental Research Letters, 8(2), 025011.

[11] Ferraro, P. J., Hanauer, M. M., Miteva, D. A., Nelson, J. L., Pattanayak, S. K., Nolte, C., & Sims, K. R. (2015). Estimating the impacts of conservation on ecosystem services and poverty by integrating modeling and evaluation. Proceedings of the National Academy of Sciences, 112(24), 7420-7425.

[12] Hanauer, M. M., & Canavire-Bacarreza, G. (2015). Implications of heterogeneous impacts of protected areas on deforestation and poverty. Philosophical Transactions of the Royal Society B: Biological Sciences, 370(1681), 20140272.

[13] Haruna, A., Pfaff, A., Van den Ende, S., & Joppa, L. (2014). Evolving protected-area impacts in Panama: impact shifts show that plans require anticipation. Environmental Research Letters, 9(3), 035007.

[14] Jiao, X., Walelign, S. Z., Nielsen, M. R., & Smith-Hall, C. (2019). Protected areas, household environmental incomes and well-being in the Greater Serengeti-Mara Ecosystem. Forest Policy and Economics, 106, 101948.

[15] Kari, F. B., Masud, M. M., Yahaya, S. R. B., & Saifullah, M. K. (2016). Poverty within watershed and environmentally protected areas: the case of the indigenous community in Peninsular Malaysia. Environmental monitoring and assessment, 188(3), 173.

[16] Le, H. D., Smith, C., & Herbohn, J. (2014). What drives the success of reforestation projects

in tropical developing countries? The case of the Philippines. Global Environmental Change, 24, 334-348.

[17] Ma, B., Cai, Z., Zheng, J., & Wen, Y. (2019). Conservation, ecotourism, poverty, and income inequality–A case study of nature reserves in Qinling, China. World Development, 115, 236-244.

[18] Manejar, A. J. A., Sandoy, L. M. H., & Subade, R. F. (2019). Linking marine biodiversity conservation and poverty alleviation: A case study in selected rural communities of Sagay Marine Reserve, Negros Occidental. Marine Policy, 104, 12-18.

[19] Miranda, J. J., Corral, L., Blackman, A., Asner, G., & Lima, E. (2016). Effects of protected areas on forest cover change and local communities: evidence from the Peruvian Amazon. World Development, 78, 288-307.

[20] Naughton-Treves, L., Alix-Garcia, J., & Chapman, C. A. (2011). Lessons about parks and poverty from a decade of forest loss and economic growth around Kibale National Park, Uganda. Proceedings of the National Academy of Sciences, 108(34), 13919-13924.

[21] Panlasigui, S., Rico-Straffon, J., Pfaff, A., Swenson, J., & Loucks, C. (2018). Impacts of certification, uncertified concessions, and protected areas on forest loss in Cameroon, 2000 to 2013. Biological conservation, 227, 160-166.

[22] Pfaff, A., Robalino, J., Herrera, D., & Sandoval, C. (2015). Protected areas' impacts on Brazilian Amazon deforestation: examining conservation–development interactions to inform planning. PloS one, 10(7), e0129460.

[23] Pfaff, A., Robalino, J., Sandoval, C., & Herrera, D. (2015). Protected area types, strategies and impacts in Brazil's Amazon: public protected area strategies do not yield a consistent ranking of protected area types by impact. Philosophical Transactions of the Royal Society B: Biological Sciences, 370(1681), 20140273.

[24] Pour, M. D., Motiee, N., Barati, A. A., Taheri, F., Azadi, H., Gebrehiwot, K., ... & Witlox, F. (2017). Impacts of the Hara biosphere reserve on livelihood and welfare in Persian Gulf. Ecological economics, 141, 76-86.

[25] Robalino, J., & Villalobos, L. (2015). Protected areas and economic welfare: an impact evaluation of national parks on local workers' wages in Costa Rica. Environment and Development Economics, 20(3), 283-310.

[26] Sims, K. R. (2010). Conservation and development: Evidence from Thai protected areas. Journal of Environmental Economics and Management, 60(2), 94-114.

[27] Sims, K. R. (2014). Do protected areas reduce forest fragmentation? A microlandscapes approach. Environmental and Resource Economics, 58(2), 303-333.

[28] Sims, K. R., & Alix-Garcia, J. M. (2017). Parks versus PES: Evaluating direct and incentive-based land conservation in Mexico. Journal of Environmental Economics and Management, 86, 8-28.

[29] Sims, K. R., Thompson, J. R., Meyer, S. R., Nolte, C., & Plisinski, J. S. (2019). Assessing the local economic impacts of land protection. Conservation Biology.（online）

[30] Yuan, J., Dai, L., & Wang, Q. (2008). State-led ecotourism development and nature conservation: A case study of the Changbai Mountain Biosphere Reserve, China. Ecology and Society, 13(2).

[31] Zhang, Q., Bilsborrow, R. E., Song, C., Tao, S., & Huang, Q. (2019). Rural household income distribution and inequality in China: Effects of payments for ecosystem services policies and

other factors. Ecological Economics, 160, 114-127.
